# Myxoma Virus-Loaded Mesenchymal Stem Cells in Experimental Oncolytic Therapy of Murine Pulmonary Melanoma

**DOI:** 10.1016/j.omto.2020.07.003

**Published:** 2020-07-06

**Authors:** Joanna Jazowiecka-Rakus, Aleksander Sochanik, Aleksandra Rusin, Agata Hadryś, Wojciech Fidyk, Nancy Villa, Masmudur M. Rahman, Ewa Chmielik, Lina S. Franco, Grant McFadden

**Affiliations:** 1 Maria Skłodowska-Curie Memorial National Research Institute of Oncology, 44-102 Gliwice, Poland; 2 Biodesign Institute, Arizona State University, Tempe, AZ 85287, USA

**Keywords:** mesenchymal stem cells, myxoma virus, melanoma, oncolytic virotherapy

## Abstract

Oncolytic viruses can target neoplasms, triggering oncolytic and immune effects. Their delivery to melanoma lesions remains challenging. Bone-marrow-derived mesenchymal stem cells (MSCs) were shown to be permissive for oncolytic myxoma virus (MYXV), allowing its transfer to melanoma cells, leading to their killing. Involvement of progeny virus was demonstrated in the transfer from MSCs to co-cultured melanoma cells. The inhibitory effect of virus on melanoma foci formation in murine lungs was revealed using melanoma cells previously co-cultured with MYXV-infected MSCs. Virus accumulation and persistence in lungs of lesion-bearing mice were shown following intravenous administration of MSC-shielded MYXV construct encoding luciferase. Therapy of experimentally induced lung melanoma in mice with interleukin (IL)-15-carrying MYXV construct delivered by MSCs led to marked regression of lesions and could increase survival. Elevated natural killer (NK) cell percentages in blood indicated robust innate responses against unshielded virus only. Lung infiltration by NK cells was followed by inflow of CD8+ T lymphocytes into melanoma lesions. Elevated expression of genes involved in adaptive immune response following oncolytic treatment was confirmed using RT-qPCR. No adverse pathological effects related to MSC-mediated oncolytic therapy with MYXV were observed. MSCs allow for safe and efficient ferrying of therapeutic MYXV to pulmonary melanoma foci triggering immune effects.

## Graphical Abstract

**Figure:**
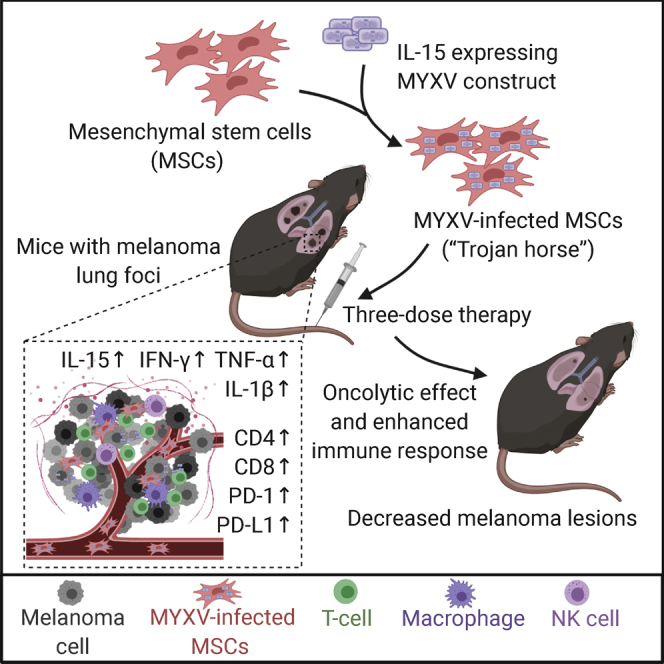

## Teaser

Myxoma virus construct carrying the IL-15 gene was used to treat experimental pulmonary melanoma in mice. The construct was delivered systemically by mesenchymal stem (“Trojan horse”) cells and triggered an oncolytic effect and antitumor adaptive immune response. This led to marked regression of lesions and makes increased survival possible.

## Introduction

The attractiveness of anti-cancer strategy using replication-competent oncolytic viruses relies on their ability to infect, replicate in, and destroy neoplastic cells and to induce antitumor immunity.^1^^,^^2^ Native oncolytic viral platforms can be engineered to reprogram expression of immunomodulatory, apoptotic, or tumor-vasculature-targeting proteins or interactions with receptors and viral gene expression cofactors.^3^^,^^4^ Oncolytic virotherapy has been gaining importance since the clinical success of talimogene laherparepvec (trade name, T-VEC or Imlygic), approved for patients with advanced melanoma in 2015. Clinical trials have investigated T-VEC as neoadjuvant monotherapy and in combination with checkpoint inhibitors also in other malignancies.^5^ Stand-alone T-VEC is only injected into accessible melanoma lesions or lymph nodes, and the distant tumor eradication rate needs to be improved. The challenge posed by poorly accessible/disseminated lesions encourages systemic delivery of oncolytic virotherapeutics.

The injection of unshielded viruses results, however, in their rapid clearance from circulation due to host defenses involving serum components, the mononuclear phagocyte system, and virion binding to plasma proteins and blood cells.^6^, ^7^, ^8^ Pre-existing neutralizing antibodies against the input virus may escalate immune responses and compromise repeated dosage. Shielding oncolytic viruses during systemic transit to tumor sites should also facilitate achievement of “viremic threshold,” crucial for the spread of therapeutic viruses.^9^

Systemic delivery strategies that shield oncolytic viruses include loading carriers with virus *ex vivo* and then re-injecting them to deliver the shielded oncolytic cargo. The carrier should support viral infection, conceal the virus from neutralizing activity during transit, and allow for tumor homing.^10^ Examples of cellular vehicles include T lymphocytes,^11^ transformed cancer cells and endothelial cells,^12^ and mesenchymal stem cells (MSCs).^10^

MSCs are multipotent stem cells from various sources (including bone marrow or adipose tissue) and display low immunogenicity due to weak expression of major histocompatibility complex (MHC) class I.^10^ They secrete pro-inflammatory cytokines in response to microenvironment cues and accumulate within tumor stroma owing to the expression of tumor-associated chemokines. MSCs create a tolerogenic microenvironment and inhibit activity of dendritic, natural killer (NK), CD8+, and CD4+ cells through the release of prostaglandins and interleukins (ILs).^10^ MSCs were used for delivering measles virus,^13^^,^^14^ herpes simplex virus,^15^ adenovirus,^16^ and vaccinia virus.^17^ Here, we used human bone-marrow-derived MSCs to deliver recombinant oncolytic myxoma virus (MYXV).

This poxvirus has an attractive safety profile; it exhibits a strict, rabbit-specific host tropism in nature and is non-pathogenic to humans or mice.^18^ It replicates selectively in immortalized/transformed non-rabbit cells, including many human cancer cell lines; normal primary human or mouse cells can abort the virus replication cycle.^19^^,^^20^ MYXV expresses immunoregulatory proteins, viroceptors, and proteins modulating macrophage and T cell functions and can be armed with transgenes.^21^ Selective MYXV replication in cancer cells results from compromised innate antiviral defense pathways (e.g., type I interferon [IFN] and tumor necrosis factor [TNF] antiviral responses)^22^ or constitutively activated signaling pathways (e.g., phosphatidylinositol 3-kinase [PI3K]/AKT).^23^ MYXV constructs were administered in acute myeloid leukemia, multiple myeloma, pancreatic and ovary cancers, glioma, and melanoma.^11^^,^^22^, ^23^, ^24^, ^25^ MYXV was also delivered by MSCs to glioblastoma *in situ*^26^ and gallbladder carcinoma,^27^ but systemic delivery of MYXV using MSCs remains unexplored.

We treated experimental murine lung melanoma with IL-15-expressing oncolytic MYXV construct delivered by human bone-marrow-derived MSCs. The crucial role of NK cells in oncoviral therapy and cytokine control of NK cells justified the use of this construct, as it was designed to strongly increase the bioavailability of IL-15 and to stimulate innate and adaptive immune responses. The construct had been reported to dramatically increase accumulation of NK cells in tumors and attenuate growth of B16-F10 melanomas.^28^

## Results

### Identity of Bone-Marrow-Derived Human MSCs

**Figure 1:**
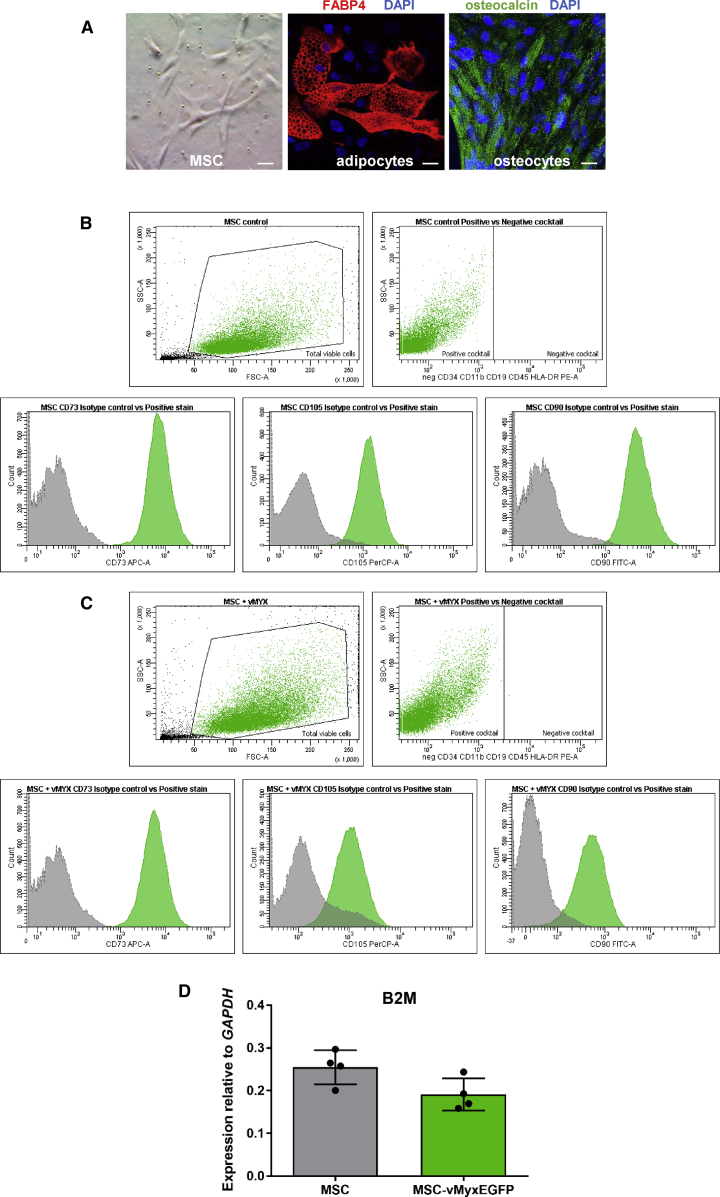
Immunophenotypic Characterization of MSCs (A) Morphology and differentiation of MSCs into adipocytes (anti-mouse FABP4 antibody) and osteocytes (anti-human osteocalcin antibody); DAPI-counterstained nuclei, magnification, 40×. Scale bars, 20 μm. (B and C) Flow cytometry plots showing the presence of MSC-associated surface markers (CD73, CD90, and CD105) and the co-occurring absence of blood cell-lineage-specific markers (CD11b, CD19, CD34, CD45, and HLA-DR on non-infected (B) and infected (with vMyx-EGFP; MOI = 10) (C) human bone-marrow-derived MSCs. Gating parameters are based on the signal of isotype IgG control probes. (D) Constitutive expression of β-2 microglobulin (B2M) in MSC-vMyx-infected cells was rendered as a ratio of target gene (*B2M*) versus reference gene (*GAPDH*) relative to expression in control mock-infected MSCs or in MSCs infected with vMyx-EGFP (MOI = 10). The data (means ± SD) were analyzed with one-way ANOVA (no significant difference).

Morphology of cultured human bone-marrow-derived MSCs was characterized along with their ability to differentiate into adipocytes and osteoblasts, as well as the presence of MSC-specific markers. MSCs show fibroblast-like morphology (Figure 1A) and differentiate into adipocytes (FABP4 protein; Figure 1A, red), or osteocytes (osteocalcin; Figure 1A, green). MYXV-infected and uninfected MSC immunophenotypes (presence of MSC-associated surface markers: CD73, CD90, and CD105; and the co-occurring absence of blood cell lineage-specific markers: CD11b, CD19, CD34, CD45, and HLA-DR) were confirmed (Figures 1B and 1C) using a research-report-based^29^ commercial kit. MYXV infection induces no significant changes in MHC class I expression on MSCs between constitutive expression of the β-2 microglobulin (B2M) gene in MYXV-infected and mock-infected MSCs 24 h post-infection (p.i.) (p = 0.0596) (Figure 1D).

### MSCs and Melanoma Cell Lines Are Permissive to MYXV Infection

**Figure 2:**
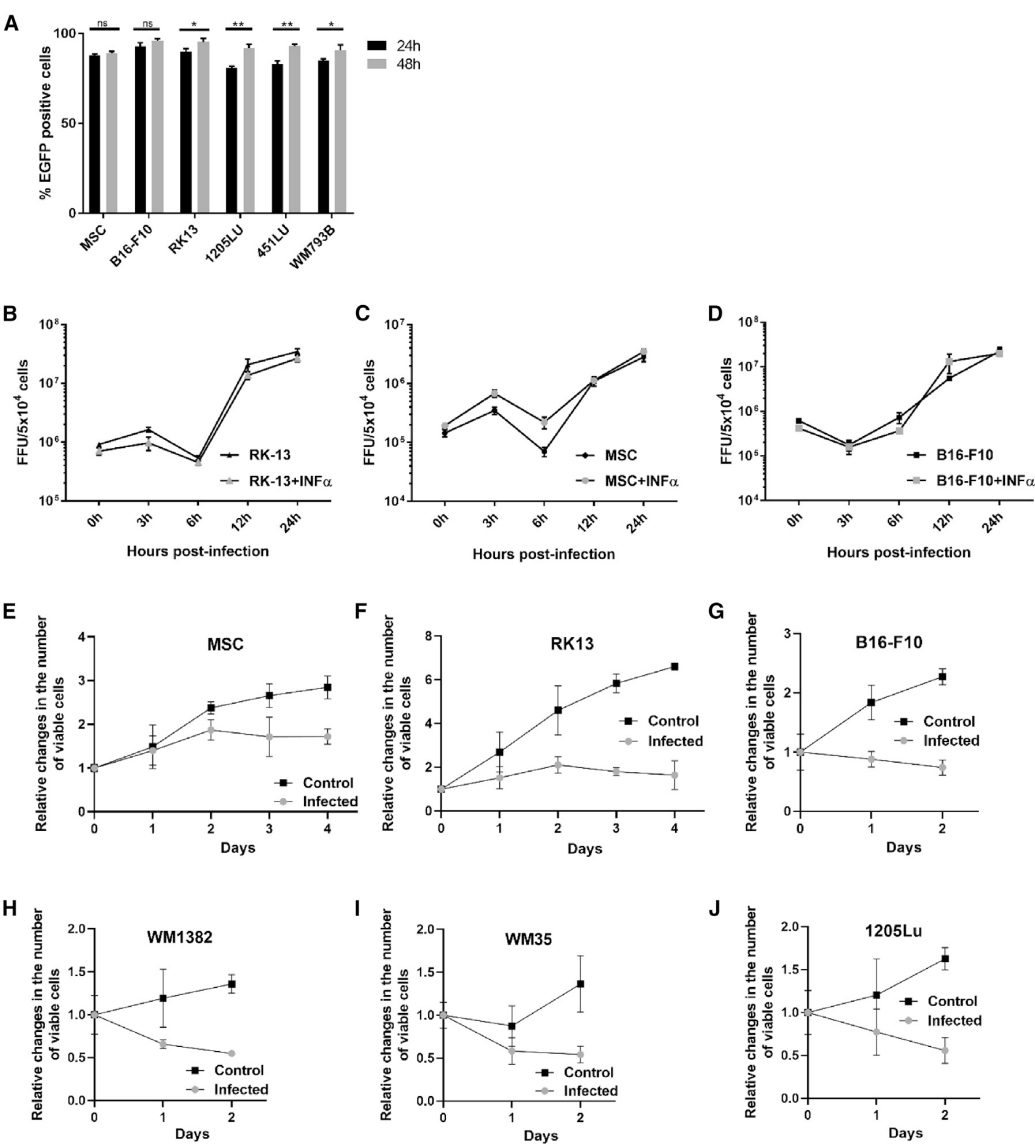
Infection of MSCs and of RK13 and Melanoma Cell Lines with vMyx-EGFP (A) Percentage of EGFP-positive cells (MSCs, B16-F10, RK13, 1205Lu, 451Lu, and WM793B), as determined by flow cytometry at 24 and 48 h p.i.; 4 × 10^5^ cells per line infected with vMyx-EGFP (MOI = 10). For data acquisition, 7-AAD emission was measured, and a region for viable cells was defined. Data (mean ± SD) were analyzed with unpaired t test; statistically significant differences are indicated (∗p ≤ 0.05; ∗∗p ≤ 0.01). ns, not significant. (B–D) Single-step growth curves (treated or not treated with 1 ng/mL IFN-α) for (B) RK13 cells, (C) MSCs, and (D) B16-F10 cells infected with vMyx-EGFP (MOI = 5; 5 × 10^4^ cells per well of a 24-well plate) and collected during a 24-h time span p.i. Virus titers determined in triplicate following serial dilution onto RK13 cells. Data (means ± SD) were analyzed with paired t test; no statistically significant difference for IFN-α challenge. (E–J) Viability of infected (MOI = 10) and non-infected (E) MSCs; (F) RK13 cells; and melanoma cells (G) B16-F10, (H) WM1382, (I) WM 35, and (J) 1205Lu was tested during indicated time spans p.i. using the alamarBlue Cell Viability Assay. The data represent the means ± SD of three independent experiments.

Analysis of flow cytometry confirmed the permissiveness of human MSCs, rabbit RK13, and murine B16-F10 as well as human 1205Lu, 451Lu, and WM793B melanomas to infection with enhanced green fluorescent protein (EGFP)-expressing MYXV construct (MOI = 10). Cells were EGFP positive (>80% at 24 h and >90% at 48 h) (Figure 2A).

### RK13, MSCs, and B16-F10 Cells Produce Infectious MYXV Progeny

Production of MYXV progeny was assessed under one-step multiplication conditions. MYXV-infected (MOI = 5) RK13, MSC, and B16-F10 monolayer cultures (treated or not treated with IFN-α) were tested. Log increase in viral titers (Figures 2B–2D) indicates more robust virus replication and production of infectious viral progeny in RK13 and B16-F10 melanoma cells than in MSCs. Presence of IFN-α did not affect MYXV ability to replicate.

### MYXV Is Cytotoxic to Melanoma Cells

MSCs remained viable (Figure 2E) after infection, and their proliferation was not remarkably reduced. On the other hand, RK13 control cells as well as B16-F10 murine melanoma and the three human melanoma cell lines (WM1382, WM35, and 1205Lu) were susceptible to killing by MYXV (Figures 2F–2J), resulting in significantly decreased percentage of viable infected cells after 48 h.

### B16-F10 Cells Targeted by MYXV-Infected MSCs Are Infected by Viral Progeny

**Figure 3:**
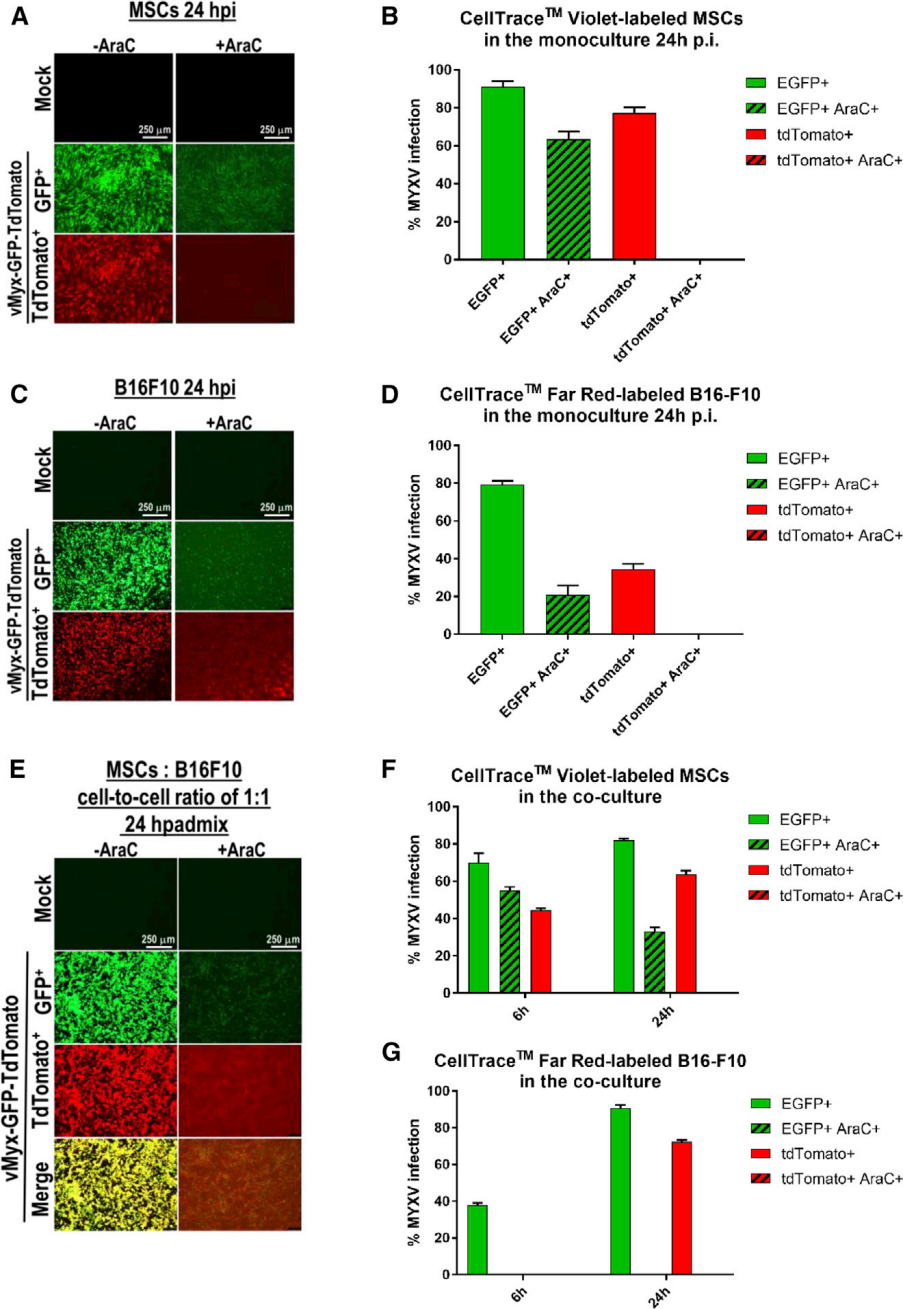
Presence of Progeny vMyx-EGFP/tdTr Reporter Construct in B16-F10 following Co-culture with Infected MSCs and Ara-C Treatment MSCs and B16-F10 cells were labeled using CellTrace Violet and CellTrace Far Red reagents, respectively, to allow *in vitro* labeling and tracing of multiple generations of cells by flow cytometry. (A–D) MSCs and B16-F10 cells separate monocultures after infection with vMyx-EGFP/tdTr and Ara-C (+ or −) treatment. (A and C) Fluorescence micrographs of infected MSCs (A) or B16-F10 cells (C). (B and D) Flow-cytometric quantitation of EGFP and tdTomato (tdTr) expression in infected MSCs (B) or B16-F10 cells (D). (E–G) MSCs pre-infected with vMyx-EGFP/tdTr, Ara-C (+ or −) treated, and subsequently co-cultured with B16-F10 cells (at a 1:1 cell-to-cell ratio). (E) Fluorescence micrographs of co-cultures after 24 h. (F and G) Flow-cytometric quantitation (6–24 h p.i.) of EGFP and tdTomato expression in MSCs (F) and B16-F10 cells (G). Scale bars, 250 μm. The data represent means ± SD of three independent experiments.

Contribution of input versus progeny to MYXV transfer from MSCs to B16-F10 was studied using MSCs stained with CellTrace Violet and B16-F10 cells stained with CellTrace Far Red reagent. For infection (MOI = 10) the vMyx-EGFP/tdTr tandem construct was used, allowing EGFP expression at both the early and late infection stages and tandem dimer Tomato red fluorescent protein (tdTr) expression only at the late infection stage. Addition of cytarabine (also termed Ara-C; inhibitor of late gene expression) to infected MSCs or B16-F10 monocultures (Figures 3A and 3C), indeed, led to the inhibition of late MYXV gene expression at 24 h p.i. (no red fluorescence). Flow cytometry data confirmed the inhibition in MSCs (Figure 3B) and B16-F10 cells (Figure 3D). Ara-C added to the co-culture of infected MSCs and B16-F10 cells (Figure 3E) led to strongly inhibited late MYXV gene expression after 24 h (no red fluorescence). Flow cytometry data for the Ara-C-treated co-culture is indicative of late viral gene expression inhibition in MSCs (Figure 3F) and B16-F10 melanoma cells (Figure 3G). EGFP expression in melanoma cells following Ara-C treatment of co-cultures was diminished but not eliminated. This implies MYXV progeny contribution to the infection of targeted melanoma cells.

### MYXV Infection Spreads from MSCs to Melanoma Cells during *In Vitro* Co-culture

Live-cell imaging (3–48 h p.i.) using time-lapse fluorescence microscopy (Video S1) revealed cell-to-cell contacts developing during the co-culture of vMyx-EGFP-infected MSCs (green) with monomeric red fluorescent protein (mRFP)-expressing B16-F10 melanoma cells (red). After 24 h, yellow-orange fluorescence (overlap) is present in melanoma cells reflecting transfer of MYXV progeny; further transfer from infected and destroyed B16-F10 cells to uninfected ones is also seen. Following contact with B16-F10 cells and transfer of MYXV via cell-to-cell contacts, the infected MSCs remained viable. See Video S1 for details.

Video S1. Co-culture of MYXV-Infected MSCs with Melanoma Cells Time-lapse (3-48h) fluorescence microscopy of vMyx-EGFP-infected (MOI=10) MSCs (green fluorescence) cocultured (1:2 cell/cell ratio) with mRFP-expressing B16-F10 (red fluorescence). Cell-to-cell contact (visible from ca. 3-h time point) between MSCs and melanoma cells enabling cross-infection; yellow-orange fluorescence (overlay) from infected melanoma cells visible besides red and green signals (magn. 10×, scale bar 50 μm); (AVI file: 97.4 MB).

### Injection of B16-F10 Melanoma Cells Co-cultured with MSCs Pre-infected with MYXV Inhibits Growth of Pulmonary Tumors

**Figure 4:**
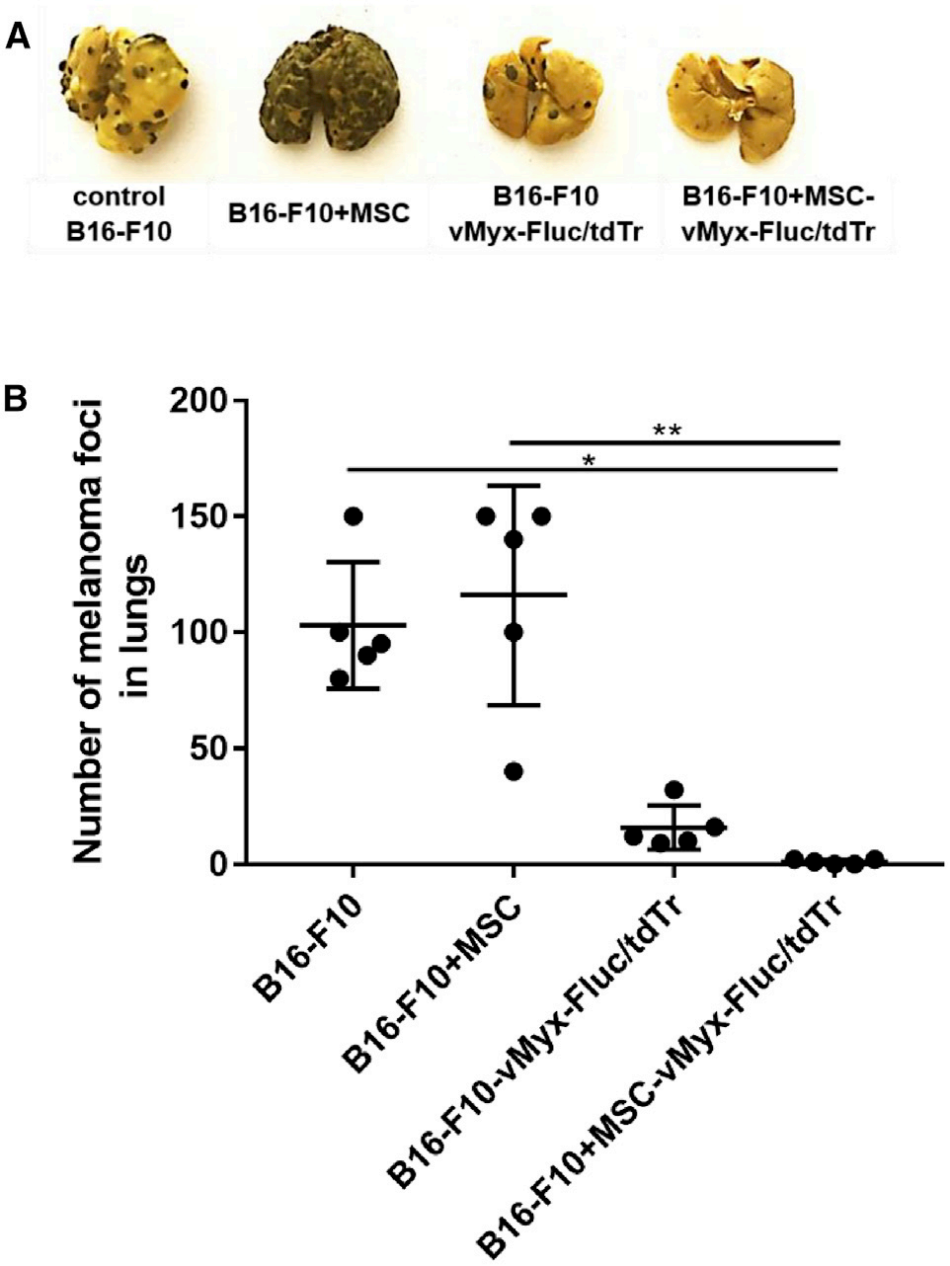
Inhibition of Pulmonary Foci Formation after i.v. Injection of MYXV-Infected B16-F10 Melanoma Cells (A and B) Visible B16-F10 melanoma pulmonary foci (A) and quantification (B) 3 weeks post-injection of B16-F10 cells (control), B16-F10 cells previously co-cultured with MSCs (B16-F10+MSC), B16-F10+MSCs pre-infected with MYXV construct (B16-F10+MSC-vMyxFluc/tdTr), or B16-F10+MSC or B16-F10 cells pre-infected with MYXV (B16-F10-vMyx-Fluc/tdTr). Data (mean ± SD) were analyzed with one-way ANOVA (n = 5); statistically significant differences are indicated (∗p ≤ 0.05; ∗∗p ≤ 0.01).

Mice injected intravenously (i.v.) with B16-F10 melanoma cells previously co-cultured with pre-infected MSCs and mice injected with B16-F10 melanoma cells pre-infected directly with MYXV both showed (Figures 4A and 4B) remarkably reduced (ca.100-fold) numbers of pulmonary foci upon dissection 3 weeks later (one-way ANOVA, p < 0.001). The number of foci in the lungs of mice inoculated with B16-F10 cells co-cultured with “empty” MSCs was similar to that in untreated control, but the macroscopic appearance of lungs most likely implies larger lesions. The number of foci in the MSC-only group is in stark contrast to that of mice inoculated with melanoma cells co-cultured with MSCs infected with MYXV (see Discussion).

### MYXV-Mediated Bioluminescence Studies

**Figure 5:**
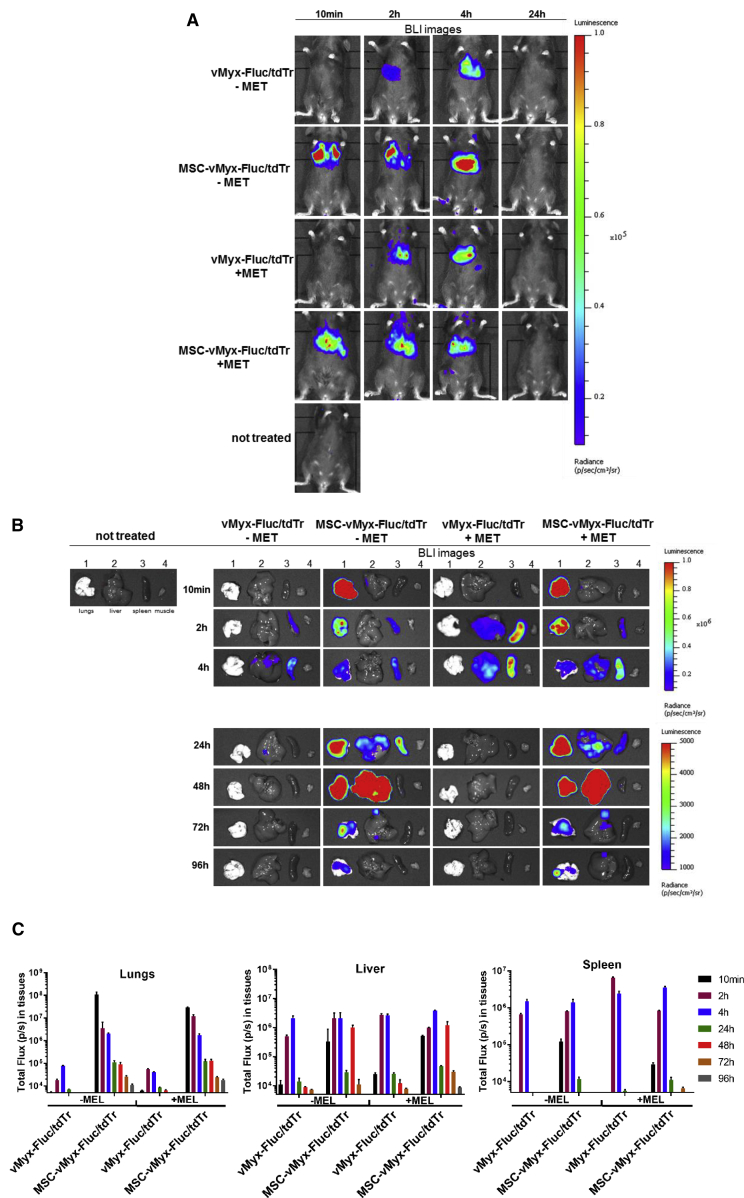
Effect of Systemically Injected MYXV Construct on Biodistribution in Mice (A and B) Bioluminescence imaging (BLI) of unchallenged (−MEL) and lung melanoma-bearing (+MEL) mice at various time points post-injection of MSC-shielded (MSC-vMyxFluc/tdTr) or unshielded reporter MYXV construct (vMyxFluc/tdTr). (A) Intact mice; (B) dissected organs (1, lungs; 2, liver; 3, spleen; and 4, muscle). BLI was expressed as radiance (photons/s/cm^2^/sr). Different radiance scales are shown to cover the whole span of bioluminescence. (C) ROI-based analysis of photon flux in dissected tissues (n = 3). The data represent mean ± SD of two independent experiments.

Bioluminescence data reflected the distribution of i.v. injected MYXV construct (unshielded or MSC shielded) expressing luciferase (vMyx-Fluc/tdTr construct encodes firefly luciferase protein [Fluc] and tdTR; Fluc under the poxvirus synthetic early/late promoter and tdTr under a poxvirus p11 late promoter, respectively). Bioluminescence signal in the lungs, following a single i.v. injection of shielded MYXV, peaked within minutes, later fading away within ca. 4 days. In contrast, bioluminescence signal following injection of the unshielded MYXV construct was not detected in the lungs at all after 2 h; instead, it was present only in the liver and spleen and faded away. At the 24-h time point, the unshielded MYXV construct was not detected in any organ at all as opposed to the MSC-shielded construct, the presence of which in the lungs was detected in addition throughout the examined time span (96 h post injection). No bioluminescence signal in muscle tissues from any tested group was observed. Region of interest (ROI) data assigned to different organs (Figure 5C) show double signal intensity for lungs at 48 h post injection (+MEL versus −MEL mice). Bioluminescence signal for MSC-protected MYXV is also high in the livers of +MEL and −MEL animals at 48 h post injection (see Discussion).

### Systemic Therapy Eliminates Experimental Melanoma Foci from Murine Lungs

**Figure 6:**
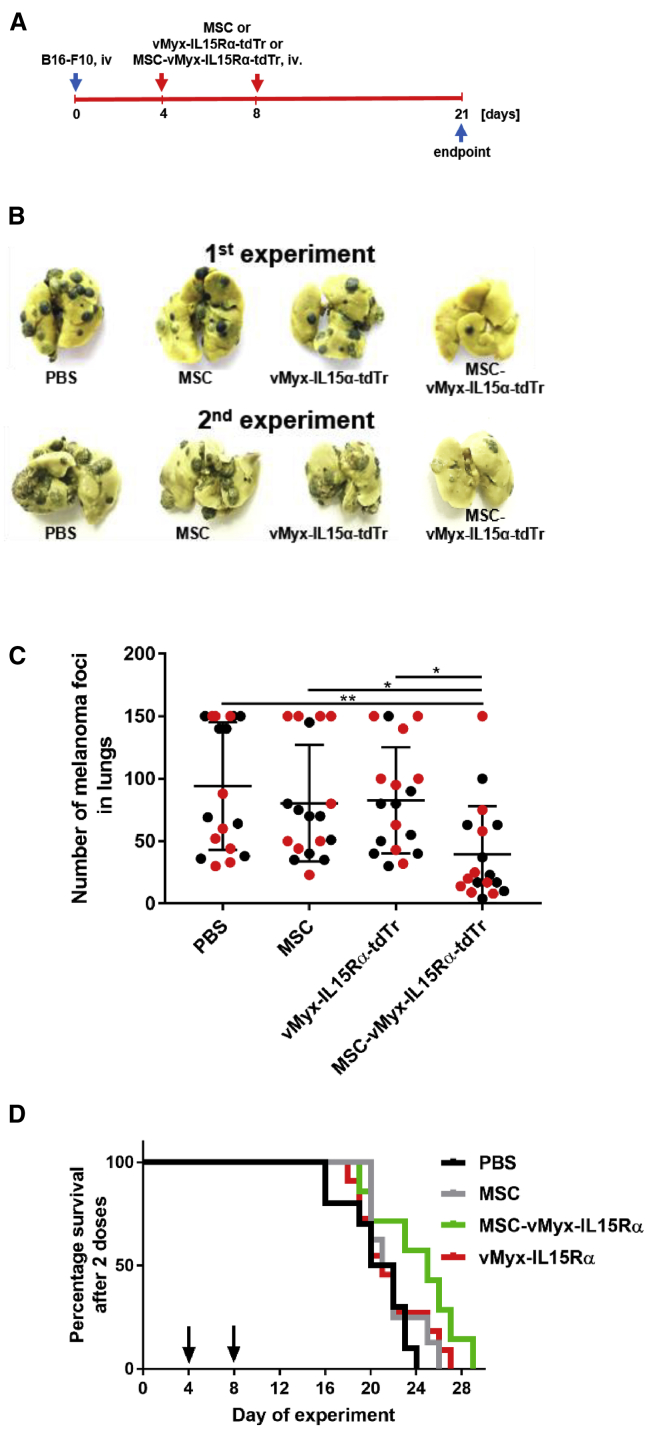
Therapy of Experimental Pulmonary Melanoma with two Administrations of MYXV Construct Two independent experiments were performed. C57BL6 mice (n = 9) with previously induced B16-F10 lung foci were i.v. injected on days 4 and 8 with IL-15-expressing MYXV construct (vMyxIL15Rα-tdTr), either MSC shielded or unshielded, or with MSCs alone or with PBS. (A) Timeline of experiment. (B) Visible melanoma foci in excised lungs after 21 days. (C) Quantification of foci (black and red dots indicate 1st and 2nd experiments, respectively). The data (mean ± SD) were analyzed with one-way ANOVA; statistically significant differences are indicated (∗p ≤ 0.05; ∗∗p ≤ 0.01). (D) Mouse survival (n = 7–11); log-rank (Mantel-Cox) test, p = 0.1192 (no significant difference).

Mice with experimentally established pulmonary melanoma foci were subjected to treatment with MSC-shielded MYXV construct encoding IL-15 (vMyx-IL15Rα-tdTr expressing IL-15 complex with the α subunit of its receptor and tdTR under a poxvirus synthetic early/late promoter). Two independent experiments were performed involving administration (days 4 and 8 after tumor inoculation) of the therapeutic system (Figure 6A). Gross inspection of dissected lungs at the conclusion of the experiment (Figure 6B) revealed, on average, a 2.5-fold reduction of the number of pulmonary foci (Figure 6C) as compared to the B16-F10 untreated control group (p = 0.0028) and a 2-fold reduction as compared to the unshielded virus group (p = 0.0263) and MSC group (p = 0.0387), suggesting positive response to the treatment. Difference in survival (p = 0.1192) was not significant for the therapeutic scheme applied twice (Figure 6D). However, with the same system administered three times (at days 4, 8, and 12 post-tumor inoculation), extension of survival (Figure 8I) became borderline significant (p = 0.0485).

### MYXV Delivery Raises NK Cell Level in Peripheral Blood and Lungs and Triggers Adaptive Immune Response

**Figure 7:**
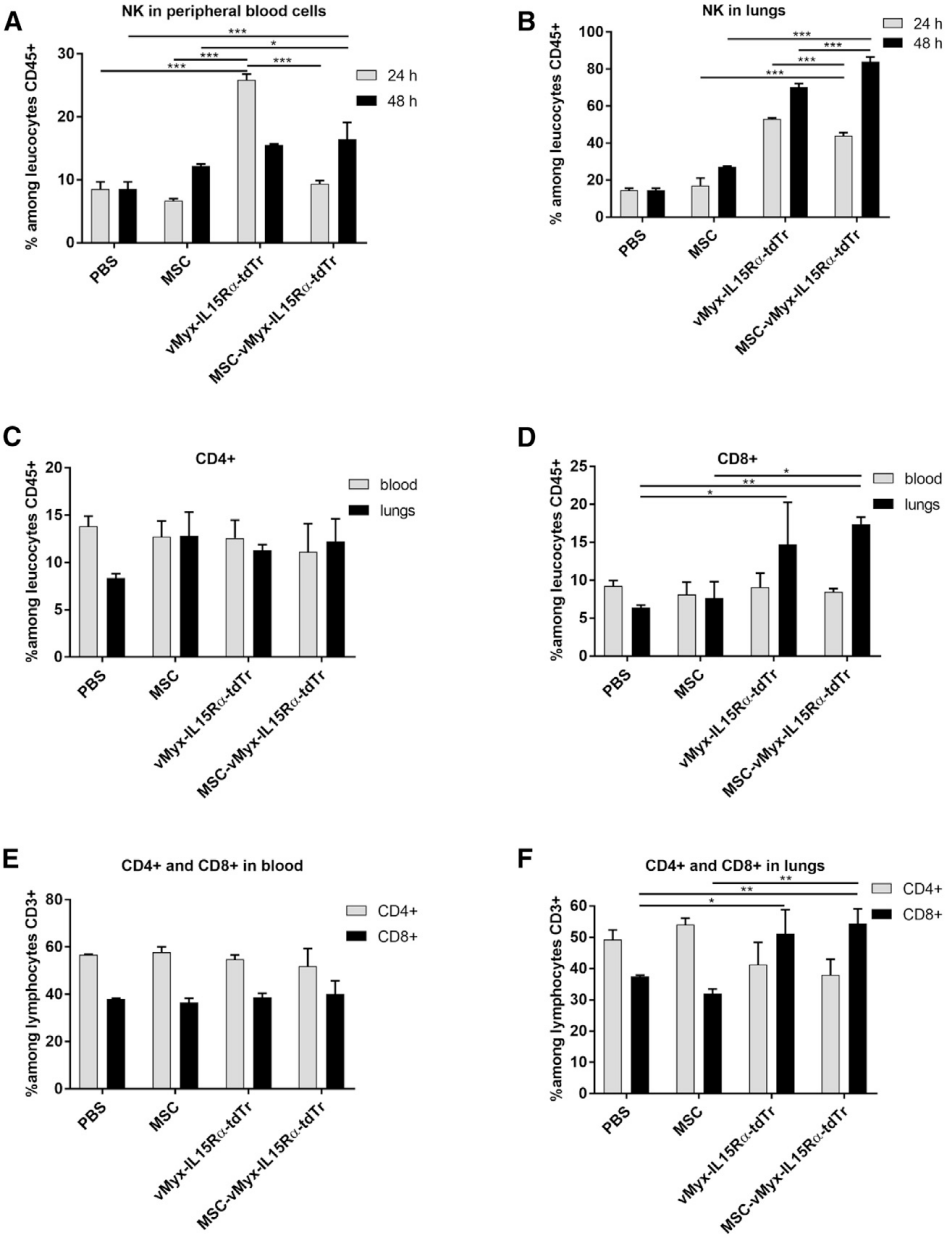
Immune Response following Therapy of Experimental Pulmonary Melanoma with Two Administrations of MYXV Construct C57BL6 mice (n = 3) with previously induced B16-F10 lung foci were i.v. injected on days 4 and 8 with IL-15-expressing MYXV construct (vMyxIL15Rα-tdTr), either MSC shielded or unshielded, or with MSCs alone or PBS. (A and B) Flow cytometry data show NK cell percentages in (A) peripheral blood and (B) lungs at 24 h and 48 h post-treatment. (C–F) CD4+ and CD8+ cell percentages in peripheral blood and lungs at the experiment endpoint (21^st^ day). (C) CD4+; (D) CD8+; (E) CD4+ and CD8+ in blood; and (F) CD4+ and CD8+ in lungs. The data (mean ± SD) were analyzed with one-way ANOVA; two independent experiments were performed, and statistically significant differences are indicated (∗p ≤ 0.05; ∗∗p ≤ 0.01; ∗∗∗p ≤ 0.001).

Early immune response following i.v. administration of unshielded or MSC-shielded MYXV construct expressing IL-15 was evidenced by flow cytometry (Figures 7A and 7B). Levels of NK, CD4+, and CD8+ cells were determined in peripheral blood and single-cell suspensions derived from lungs of mice with established B16-F10 pulmonary melanoma foci. The percentage of NK cells in blood (Figure 7A) at the 24-h time point was 2.8-fold higher for unshielded MYXV construct than for shielded MYXV (p < 0.0001), clearly suggesting an early innate immune response to unshielded MYXV expressing the IL-15 fusion protein. For the shielded MYXV construct, NK cell percentage did not differ significantly from controls. At the 48-h time point, however, in the case of unshielded MYXV, the percentage of NK cells decreased considerably, not differing significantly from that for the shielded MYXV construct or controls. In the lungs (Figure 7B), the NK cell percentage at 24 h post injection for the unshielded MYXV was also higher than for the shielded MYXV (p = 0.0054), but, more importantly, both were >3-fold higher than the controls. At 48 h post injection, the NK cell percentage in the lungs for MSC-shielded MYXV construct was almost doubled (compared to the 24-h time point), which may be associated with transfer of virus between MSCs and melanoma cells. The adaptive antitumor immune response was examined (Figures 7C–7G) after 3 weeks (therapy endpoint). The percentage of CD4+ helper cells among leukocytes (Figure 7C) remained generally unchanged for both blood and lungs. Likewise, cytotoxic tumor-infiltrating CD8+ cells among leukocytes (Figure 7D) were present at similar percentages in blood for all treatment groups. For lung tissue, mice treated with either unshielded MYXV or MSC-shielded MYXV showed, in contrast, a significant increase (almost 2.5-fold) of CD8+ T cells (p < 0.05). Among CD3+ lymphocytes, the level of CD4+ and CD8+ cells in blood remained unchanged (Figure 7E) for all groups; however, in the lungs of mice treated with shielded or unshielded construct, the percentage of CD4+ was decreased (p < 0.05), and that of CD8+ cells was increased (p < 0.05) (Figure 7F).

### MYXV Delivery by MSCs Raises Lung Expression of Genes Affecting Adaptive Immune Response

**Figure 8:**
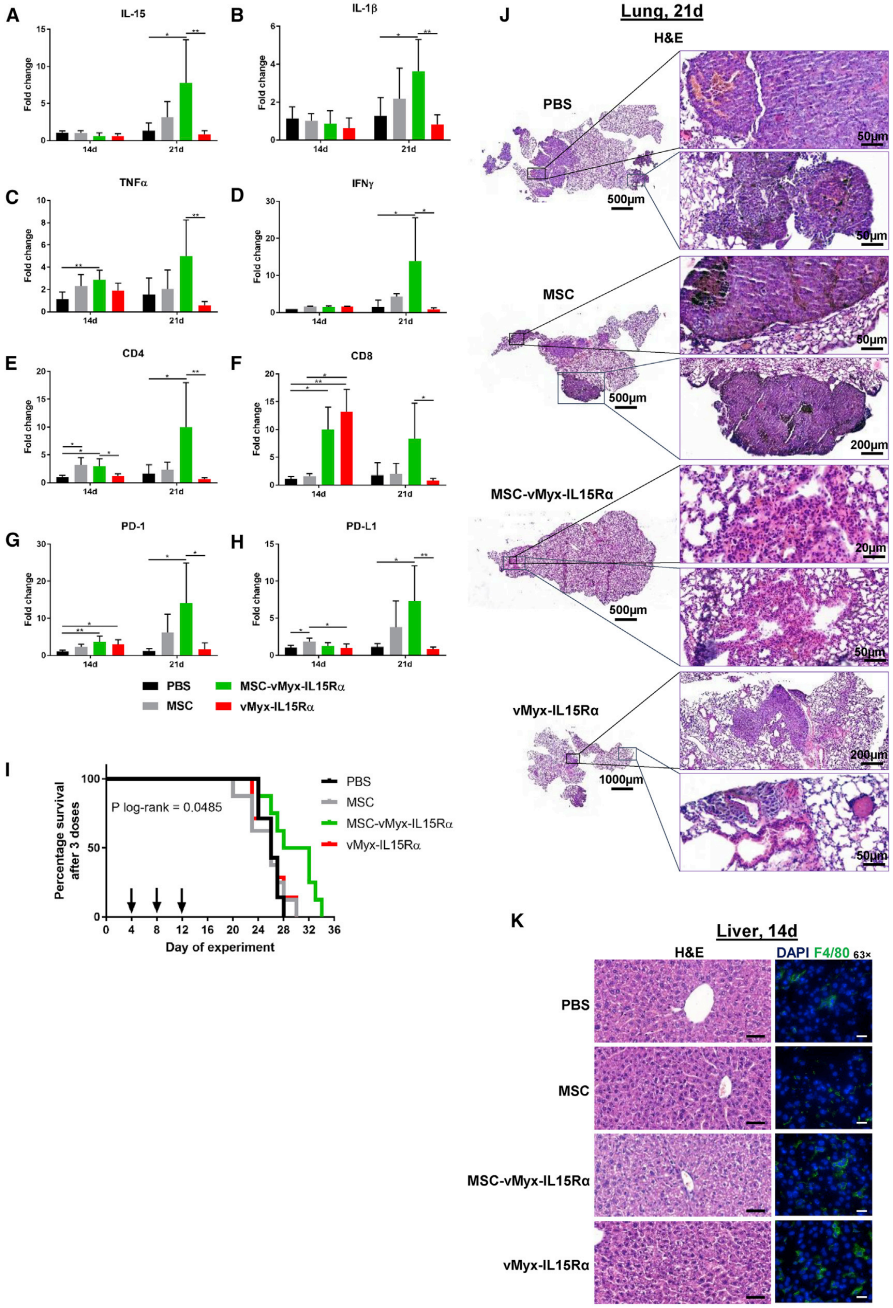
Therapy of Experimental Pulmonary Melanoma with Three Administrations of MYXV Construct: Immune Response, Survival, and Histopathology C57BL6 mice with previously induced B16-F10 lung foci were i.v. injected on days 4, 8, and 12 with IL-15-expressing MYXV construct (vMyxIL15Rα-tdTr), either MSC shielded or unshielded, with MSCs alone, or with PBS. (A–H) RT-qPCR and gene expression analysis from lung sections for (A) IL-15, (B) IL-1β, (C) TNF-α, (D) IFN-γ, (E) CD4, (F) CD8, (G) PD-1, and (H) PD-L1. Changes in the gene expression were rendered as a ratio of target gene versus reference gene (*GAPDH*) relative to expression in control samples. The data (mean ± SD) were analyzed with one-way ANOVA (statistically significant differences are indicated; ∗p ≤ 0.05; ∗∗p ≤ 0.01). (I) Mouse survival (n = 7–8); log-rank (Mantel-Cox) test, p = 0.0485. (J) Histological appearance of representative H&E-stained lung sections from each treatment group (left panel: scale bars, 500–1,000 μm; right panel: scale bars, 20–200 μm). (K) Histological appearance of representative liver sections for each treatment group: H&E-stained sections (left panel: scale bars, 20 μm) and anti-F4/80 antibody-stained fixed frozen sections (right panel: scale bars, 20 μm).

Gene expression profiles of several genes encoding pro-inflammatory cytokines as well as CD4, CD8, PD-1, and PD-L1 were quantified by RT-qPCR in lung tissue collected from mice subjected to three-dose experimental therapy with unshielded or MSC-shielded IL-15Rα-tdTr construct (see Materials and Methods). Changes were analyzed at two time points (days 14 and 21) between the two experimental groups (MSC-vMyxIL15Rα-tdTr versus vMyxIL15Rα-tdTr). No changes in IL-15 expression were found (Figure 8A) between the two experimental groups after 14 days. However, on the 21^st^ day, IL-15 was upregulated, and the difference between both groups was significant (p = 0.0023). Expression of IFN-γ (Figure 8B) was upregulated on the 21^st^ day, and a significant difference exists between the two experimental groups (p = 0.0441). For TNF-α (Figure 8C), as for other inflammatory cytokines examined, strong upregulation was also detected on the 21^st^ day with significant differences in expression (p = 0.0070) between experimental groups. As for the other inflammatory cytokines, IL-1β expression on the 21^st^ day (Figure 8D) was upregulated with significant difference between the experimental groups (p = 0.0064). Concerning infiltration of immune effector T cells, the gene expression for markers of both CD4 (Figure 8E) and CD8 (Figure 8F) was upregulated on the 21^st^ day of the experiment, with significant difference between the two treatment groups (p = 0.033). The expression of CD8 was even more elevated in both experimental groups on the 14^th^ day, with especially high value for the unshielded virus (p = 0.0014). Upregulation for PD-1 (Figure 8G) and its ligand, PD-L1 (Figure 8H), was observed after 21 days; in both groups, the differences between experimental groups were significant (p = 0.0183 and p = 0.0027, respectively). RT-qPCR analysis of the expression profile of several genes (encoding pro-inflammatory cytokines as well as CD4, CD8, PD-1, and PD-L1) was examined following therapy administered on days 4, 8, and 12 post-tumor inoculation (Figure 8I). Tumor tissues were collected on days 2 and 9 after the last treatment (i.e., on days 14 and 21 of the experiment, respectively).

### Effect of MYXV-Loaded MSCs on Lungs and Liver

Histopathologic sections (Figures 8J–8K) of lung and liver tissues isolated following the three-dose therapy were examined by an experienced pathologist. H&E-stained lung sections (Figure 8J) from control mice indicate cellular heterogeneity of melanoma foci, with the presence of both epithelioid and spindle cells with high melanin content, large hemorrhage areas in lung parenchyma, congested alveoli capillaries and septa, and erythrocytes in the alveolar lumen. Lung sections from mice that were administered unshielded virus show even more disperse and heterogeneous cancer foci, large vessels, and preserved columnar bronchial epithelium, whereas lung sections from mice treated with shielded oncolytic virus show sclerotized septa and hyperemic areas probably representing healed tumor foci, with no hemorrhage areas present. H&E-stained liver sections (Figure 8K) seem to indicate that, 48 h post-treatment with shielded MYXV construct, there are neither obvious signs of melanoma infiltrations in liver parenchyma nor signs of inflammatory condition present (no lymphoid or plasmacytic infiltrates in portal tracts, no piecemeal necrosis, no cytoplasm granularity, etc.). Immunostaining of the macrophage marker F4/80 suggests (Figure 8K, right column) increased presence of Kupffer cells (green) in liver parenchyma in mice injected i.v. with unshielded MYXV construct.

## Discussion

Oncolytic viruses are emerging as a powerful anti-cancer tool, causing destruction of neoplastic cells and triggering antitumor immune response. Limitations of T-VEC therapy for unresectable melanoma encourage the search for efficient i.v. delivery to poorly accessible lesions (e.g. lung or brain metastases).^30^ Systemic delivery would allow combinations with checkpoint inhibitors,^5^ gene therapies,^31^ or targeted chemotherapies.^32^ High titers of oncolytic virus at tumor sites are critical for therapy success; thus, clearance of i.v. injected virus is a challenge.^33^ MSCs have been proposed to shield oncolytic viruses in bloodstream transit.^32^ MSCs act in synergy with oncolytic viruses, releasing pro-inflammatory cytokines.^16^^,^^34^ On a cautionary note, MSCs were reported to create a tumor-tolerogenic microenvironment by via prostaglandins and ILs and by decreasing the activity of immune cells.^10^ Syngeneic and allogeneic MSCs were reported to induce in mice systemic immune response and intratumoral leukocyte infiltration.^35^ Efficacy of MSCs was demonstrated in a phase I ovarian cancer clinical trial.^14^ We show human bone-marrow-derived MSCs to be suitable for i.v. delivery of MYXV constructs to immunocompetent mice with experimentally induced pulmonary melanoma lesions. MYXV delivery via adipose-tissue-derived MSCs to locations distant from the injection site^26^ and tumor homing^27^ was reported for MYXV-carrying MSCs.

MSCs used in here retained after infection hematopoietic stem cell markers (lack of CD45, CD34, CD11b, CD19, and HLA-DR and presence of CD105, CD73 and CD90). No changes in MHC class I expression were found after infection. The melanoma cell lines we tested were permissive to MYXV yielding infectious viral progeny (see B16-F10), and cultures underwent destruction within 2 days. IFN-α had no significant effect on virus proliferation.^36^ Replication of MYXV in MSCs was considerably less robust. Modest permissiveness and largely unaffected viability p.i. suggest suitability of MSCs for *in vivo* delivery of MYXV.

Infected MSCs transfer MYXV to co-cultured B16-F10 cells via cell-to-cell contact, on which the infected B16-F10 cells change morphology and release infectious viral particles. The contribution of input and progeny MYXV to virus transfer from MSCs to co-cultured B16-F10, examined with Ara-C and MYXV construct allowing early and late EGFP expression but only late tdTr expression,^37^ indicated that, at least during the first 24-h period, the transfer via cell-to-cell contact is exclusively active (i.e., progeny virus only); no passive transfer takes place, unlike in the case of primary human T cells, which transfer MYXV passively.^11^ The difference probably relates to how quickly specific donor cells internalize input virus from the cell surface.

We documented significant inhibition of melanoma foci formation in lungs if injected B16-F10 cells had been pre-infected with MYXV (either directly or via co-culture with MYXV-infected MSCs). Shielding of the virus by MSCs does not abrogate MYXV inhibitory effect *in vivo*. Challenging mice with co-cultures of melanoma cells with MYXV-infected MSCs led to strong inhibition of melanoma development in lungs. The finding why B16-F10 cells that had been co-cultured with uninfected MSCs seemed to enhance the tumorigenic effect, suggestive of a tumor-tolerogenic microenvironment^38^^,^^39^ and contribution to metastasis,^40^^,^^41^ and despite that the number of foci formed did not differ from that of foci established by unchallenged melanoma cells,^10^ could be attributed, at least in part, to enhanced angiogenesis. Proliferating tumor cells form an inflammatory milieu (“wound that never heals”) in which MSCs exhibit tissue repair functions and support angiogenesis, which contributes to the growth of the existing tumor foci.^40^^,^^42^, ^43^, ^44^

After i.v. injection, MSCs appear early and at highest densities in the lungs^45^ due to the predominant “first-pass” effect. MYXV-derived bioluminescence signal persisted in the lungs for 96 h but only when MSC delivered. This suggests MSC confinement to the lungs, transmission of virus to melanoma cells, or both. Fading signal intensity in the case of liver and spleen precludes the presence of tumor foci. Surprisingly high (albeit transient) liver signals after 48 h post-injection of MYXV-loaded MSCs in both +MEL and −MEL mice was accompanied by the lack of liver tumor foci upon macroscopic examination and no deviations from normal liver histology. These findings suggest concerted removal of MYXV from lungs and transfer to the liver for disposal as a potential explanation, but the virus origin must await further experimentation in the future. In agreement with rapid clearance after i.v. injection, the unshielded MYXV construct caused rapid increase in the percentage of NK cells in blood and failed to accumulate in the lungs. Incubating MSCs with MYXV for 1.5 h prior to administration did not change the lung signal when compared to 24-h incubation (data not shown). This does not preclude the likely therapeutic benefits of MSCs with just adsorbed MYXV owing to simple dissociation.^12^

The MSC-shielded MYXV construct clearly blunted the early NK cell response, whereas in the lungs, the increased percentage of NK cells correlates well with bioluminescence at 48 h only for the MSC-protected virus. This might reflect the release of MYXV cargo from lung-settled MSCs and/or from virus-infected melanoma cells. For therapy, we used MSCs infected with IL-15 fusion protein-expressing MYXV. IL-15 is an NK- and T cell-activating cytokine and potentiates influx of NK cells and CD8+ T cells into the lungs. The cytokine fusion to a subunit of its receptor was reported to increase bioavailability and immune response.^28^ Despite decreased number of lung lesions, the observed animal survival for two-round oncolytic therapy did not reach statistical significance. On the other hand, the three-round oncolytic therapy of melanoma pulmonary-lesion-bearing mice showed increased (borderline) animal survival (p = 0.0485), upregulated expression of genes encoding pro-inflammatory cytokines, and markers for infiltrating immune effector T cells (CD4 and CD8), which points to the activation of antitumor immune response. Although the latter seems counteracted by upregulation of PD-1 and its ligand, PD-L1, melanoma would be amenable to the use of checkpoint inhibitors.

Microscopic examination revealed disperse heterogeneous melanoma lesions in lungs and large hyperemic areas with congested alveoli in both untreated and unshielded MYXV-treated groups, whereas sclerotized septa and no hyperemic areas in lung sections of MSC-MYXV-treated mice probably represent healed tumor foci. A similar treatment approach did not elicit signs of an inflammatory condition or melanoma infiltrates in the liver parenchyma. Increased presence of Kupffer cells in livers from the unshielded virus-treated group suggests benefits from virus shielding strategy.

Optimizing further the administration of the proposed oncolytic therapeutic system is needed. Treatment of tumors at other anatomical locations might be feasible with achieved MSC passage to arterial circulation following transit through the lung microvasculature, e.g., via MSC size reduction and saturation of MSC adhesion to the vascular endothelium.^46^

Increased infiltration of CD8+ cells in the lungs treated with MYXV-IL15-loaded MSCs would also benefit from adding checkpoint inhibitors^5^ or T cell agonists. Aberrant vasculature of melanomas limits the contact of tumor cells with T cells.^47^ Stand-alone oncolytic virotherapies are unlikely to yield complete tumor regression, except in “elite responders.”^48^ Thus, MSCs engineered to enhance tumor tropism, novel MYXV constructs with antiapoptotic genes,^25^ suicide genes,^49^ anti-angiogenesis factors,^50^ host response immunomodulatory cytokines,^51^^,^^52^ and combinations with nanotherapeutics^32^^,^^53^^,^^54^ are likely to be developed.^55^ We show here that infected human MSCs can transfer progeny MYXV to melanoma cells and that accumulation of IL-15-expressing MYXV in murine lung lesions following delivery by MSCs can reduce tumor burden and trigger inflow of CD8+ cells.

## Materials and Methods

### Recombinant Viruses

Four recombinant MYXV constructs (vMyx-EGFP, vMyx-EGFP/tdTr, vMyxFluc-tdTr, and vMyxIL15Rα-tdTr) were used. Constructs were derived from the wild-type Lausanne strain of myxoma poxvirus. The recombination cassettes were inserted in the intergenic region between the M135 and M136 open reading frames (ORFs). vMyx-EGFP expresses EGFP from the early/late promoter, whereas thevMyx-EGFP/tdTr tandem system allows expression of EGFP at both the early and late infection stages (early/late promoter), while tdTr is expressed only at the late infection stage (poxvirus p11 late promoter).^37^ The vMyx-Fluc/tdTr construct encodes both firefly luciferase protein (Fluc) and tdTR; Fluc under the poxvirus synthetic early/late promoter and tdTr under a poxvirus p11 late promoter, respectively.^56^ The fourth MYXV construct (vMyxIL15Rα-tdTr) expresses the IL-15 complex with an α subunit of its receptor and tdTR under a poxvirus synthetic early/late promoter.^28^

### Virus Purification and Titration

Viral strains were produced in RK13 cells (MOI = 0.1). When the cytopathic effect was visible (ca. 72 h; ±80% confluency), cells were harvested, centrifuged (1500 rpm, 10 min, 4°C), resuspended in 10 mM Tris-HCl (pH = 8), and subjected to three freeze/thaw cycles and cup sonication (2 × 1 min). Cell debris was removed by centrifugation. Homogenates containing virus were layered onto the 36% sucrose cushion and ultracentifuged (10^5^ × g /1 h, 4°C). The supernatant was removed, and the pellet was resuspended in 10 mM Tris-HCl (pH = 8) for storage at −80°C. Quantity of infectious viral particles was determined by titration on RK13 cells (4 × 10^5^ per well of a 6-well plate). After 3–4 days, fluorescent foci were counted using an inverted microscope (Leica Microsystems). Titers (focus-forming units [FFUs] per milliliter) were calculated as the number of foci × dilution.

### Cell Lines

The rabbit RK13 kidney epithelial cell line (ATCC), human (451Lu, 1205Lu, WM35, and WM793B) melanoma cell lines (Coriell Life Sciences), and the murine B16-F10 melanoma cell line (ATCC) were used. Cells were maintained in DMEM (rabbit RK13) supplemented with 10% fetal calf serum (FCS) (Sigma) or in RPMI-1640 (GIBCO) supplemented with 5% FCS (human melanomas) or 10% FCS (murine melanoma). Media contained 1% antibiotics (Sigma). Cultures grown in a 5% CO_2_ incubator at 37°C were routinely tested for mycoplasma contamination.

### MSCs

Samples of bone marrow collected from healthy donors (MSC Memorial Cancer Center, Gliwice, Poland) were diluted with Minimal Eagle’s Medium (MEM) supplemented with 10% FCS (Eurex), 1% antibiotics (Sigma), and 1% non-essential amino acids (Sigma) and transferred to a humidified incubator (37°C, 5% CO_2_). After 48–72 h, cultures were washed with PBS^−^ (without Mg^2+^ and Ca^2+^ ions) to remove non-adherent cells. Sub-confluent cultures were passaged at a 1:3 split ratio. Cells from passages 2–4 were used for further experiments. Differentiation into osteocytes and adipocytes was analyzed at passage 2, using the Human Mesenchymal Stem Cell Functional Identification Kit (R&D Systems, SC006) containing goat anti-mouse FABP4 antigen affinity-purified polyclonal antibody (adipocyte marker) and mouse anti-human osteocalcin monoclonal antibody (osteocyte marker). Cells were stained with Biotinylated Rabbit Anti-Goat IgG (immunoglobulin G) Antibody (H+L) and Texas Red Streptavidin (Vector Laboratories, BA-5000 and SA-5006, respectively) or Goat Anti-Mouse Alexa Fluor Plus 488 secondary antibody (Thermo Fisher Scientific, no. A32723). Nuclei were counterstained with DAPI (Thermo Fisher Scientific, no. 62248). Morphology of bone-marrow-derived cells was inspected using the Zeiss LSM 710 confocal workstation.

### Flow Cytometry Analysis of MSC Phenotype

To confirm isolation of MSCs, the cultured cells were collected and immediately incubated with appropriate antibodies for 20 min at room temperature (RT). Then, the cells were washed using Cell Wash Buffer (BD Biosciences) and ultimately suspended in Cell Wash Buffer. Analysis was performed on a BD FACSCanto II flow cytometer (Becton-Dickinson, Franklin Lakes, NJ, USA). Gating parameters dividing positive and negative populations were established based on the signal of isotype IgG control probes. Cells were screened using the Human MSC Analysis Kit (BD Biosciences, no. 562245) for the presence of MSC-associated surface markers (CD73, CD90, and CD105) and the co-occurring absence of blood cell-lineage-specific markers (CD11b, CD19, CD34, CD45, and HLA-DR.^29^ The phenotype of MSCs infected with vMyx-EGFP (MOI = 10) was also analyzed.

### Permissiveness of RK13, MSCs, and Cancer Cell Lines to MYXV

MSCs, RK13, and four melanoma cell lines (murine B16-F10 as well as human 1205Lu, 451Lu, and WM793B) were tested for susceptibility to MYXV infection. Cultures (4 × 10^5^ cells per well of a 6-well plate) infected with vMyx-EGFP (MOI = 10) were collected at two time points (24 and 48 h p.i.). The cells were centrifuged at 1,200 rpm for 5 min and washed twice with 2 mL PBS^−^. The cells were then resuspended in 100 μL of PBS^−^, incubated with 7-aminoactinomycin D (7-AAD; 5 μL/10^6^ cells) for 10 min to determine viability, and analyzed for enhanced GFP expression using flow cytometry (BD FACSCanto II). Non-infected cells were used as a control. For data acquisition, 7-AAD emission was measured using a long-pass filter at 670 nm, and a region for live cells was defined.

### Single-Step Growth Analysis of Viral Replication in Cell Cultures Treated with IFN-α

Cultures (triplicate wells) of RK13, MSCs, and B16-F10 cells (5 × 10^4^ cells per well of a 24-well plate) were treated for 18 h with media containing 1 ng/mL IFN-α (Cell Signaling Technology), after which cell cultures were infected with vMyx-EGFP (MOI = 5). One hour p.i., the inoculum was removed, and cells were washed with PBS^−^. Cells were further incubated (5% CO_2,_ 37°C) with fresh medium containing (or not containing) the same concentration of IFN-α as described earlier. Next, cells were trypsinized and collected immediately p.i. and at 3-, 6-, 12-, and 24-h time points. Following centrifugation (2,000 rpm, 2 min), the cells were resuspended in 200 μL hypotonic swelling buffer (5 mL of 1 M Tris-HCl [pH 8.0] and 1 mL of 1 M MgCl_2_) and frozen at −80°C. Before titration, cells were thawed and sonicated (2 × 1 min) to disaggregate virus complexes. Treated or non-treated IFN-α samples from the examined time points were titrated back on RK13 cells (4 × 10^5^ cells per well of a 6-well plate) by serial dilutions. Fluorescent foci were counted using an inverted microscope (Leica Microsystems). Titers (FFUs per milliliter) were calculated as the number of foci × the dilution factor.

For cytotoxic effects of MYXV infection: MSCs, RK13, and four melanoma cell lines (murine B16-F10 and human WM1382, WM35, and 1205Lu) were infected with vMyx-EGFP construct (MOI = 10), and their viability was evaluated with the alamarBlue Cell Viability Assay (Life Technologies) throughout indicated time spans p.i. In brief, cells were plated into 96-well plates at the density of 5 × 10^3^ cells per well. alamarBlue was added to the tested wells (in octuplicate) at the indicated time points. The fluorescent signal (excitation = 560 nm, emission = 590 nm) was measured using a Biotek plate reader. Viable cell number change during cell culture was related to the signal measured at the day of plating. The data are presented as means ± SD for individual time points.

### Co-culture of Melanoma Cells with MYXV-Infected MSCs

The retrovirus-based pLNCX2 vector (Clontech) derived from MMLV was used to obtain stable expression of mRFP in cultured B16-F10 and WM793B cells (as per manufacturer’s protocol). Cultures of MSCs infected with vMyx-EGFP (MOI = 10) were trypsinized and added to mRFP-expressing B16-F10 or WM793B cultures (1:2 cell-to-cell ratio). Infection progress was evaluated using fluorescence microscopy (Zeiss LSM 710 confocal workstation).

### Transfer of Input and/or Progeny MYXV to Melanoma Cells following Co-culture with Infected MSCs and Ara-C Treatment

Cultured MSCs labeled using the CellTrace Violet Cell Proliferation Kit (Invitrogen) were infected with vMyx-EGFP/tdTr (MOI = 10) for 1 h (5% CO_2_, 37°C). The vMyx-EGFP/tdTr tandem system allows expression of EGFP at both early and late infection stages (early/late promoter), while tdTr is expressed only at the late infection stage (poxvirus p11 late promoter).^37^ Unbound virus was washed away and 50 μg/mL of Ara-C solution (Ara-C is an inhibitor of poxviral DNA replication and late gene expression of MYXV) was added to evaluate the infection. After 24 h, B16-F10 melanoma cultures were stained with CellTrace Far Red reagent (Invitrogen), and, following trypsinization, melanoma cells were added to the Ara-C-treated or untreated MSC cultures (1:1 cell-to-cell ratio). The co-culture was incubated further (5% CO_2_, 37°C) up to 48 h. Infection was evaluated using fluorescence microscopy (Leica) and flow cytometry (BD FACSCanto).

### Animal Care

Six- to-eight-week-old C57BL/6 female mice (Charles River Laboratories, Wilmington, MA, USA) were used. All animal procedures were performed in accordance with European Union (EU) law, after approval by the Local Ethics Committee, Medical University of Silesia in Katowice, Katowice, Poland.

Animals (18–22 g) were kept in HEPA-filtered IVC System cages (Allentown Caging Equipment, Allentown, NJ, USA) under a 12-h:12-h dark:light cycle and received a pathogen-free standard diet (Altromin 1314, Lage, Germany) and water *ad libitum*. All efforts were made to minimize animal suffering. After injection of melanoma cells, animal health was monitored daily. Only single animals from control groups reached termination criteria. Euthanasia was conducted by cervical dislocation.

### Systemic Administration of MYXV-Preinfected Melanoma Cells

B16-F10 cells were co-cultured *in vitro* for 24 h with MSCs that had been pre-infected (MOI = 10) using vMyxFluc/tdTr (2:1 cell-to-cell ratio) or with non-infected MSCs. Unchallenged mice were then injected (using 100 μL PBS^−^) either with 3 × 10^5^ cells from such co-cultures or with 2 × 10^5^ B16-F10 cells infected using unshielded vMyxFluc/tdTr (MOI = 10). After 3 weeks, mice were sacrificed, and their lungs were excised and fixed in Bouin’s solution. Lungs were dissected into individual lobes, and visible tumor foci were counted using a stereomicroscope.

### Bioluminescence Imaging (BLI) of MYXV Presence in Mice

Melanoma pulmonary foci were induced in C57BL6 mice by tail injection (day 0) of 2 × 10^5^ B16-F10 cells (in 100 μL PBS^−^). Recipient mice with melanoma lesions growing in lungs for 9 days (+MEL) or unchallenged mice (−MEL) were injected (day 9) with a single dose of MSCs (5 × 10^5^/100 μL PBS^−^) loaded (for 24 h) with either vMyxFluc/tdTr construct or with unshielded vMyxFluc/tdTr (5 × 10^6^ FFUs). BLI of luciferase reporter gene expression was performed using the Lumina IVIS Imaging System (PerkinElmer). At time points ranging from 10 min to 96 h post-treatment, mice (n = 3) were injected (in the neck scruff) with 1.5 mg d-luciferin (Promega, Madison, WI, USA), and BLI was performed. Measurements in intact animals were immediately followed by examination of dissected organs (lungs, liver, spleen, and leg muscle).

### Therapy of Mice Bearing Experimental Pulmonary Melanoma Foci

Melanoma lung foci in recipient C57BL/6 mice were established (day 0) by i.v. injection of B16-F10 (2 × 10^5^ cells per 100 μL PBS^−^). For therapy, mice were injected (on days 4 and 8 for two-dose therapy or on days 4, 8, and 12 for three-dose therapy) with MSCs (5 × 10^5^ cells per 100 μL PBS^−^), loaded (for 24 h) with IL-15-expressing vMyxIL15Rα-tdTr construct (5 × 10^6^ FFUs/100 μL PBS^−^) or unshielded vMyxIL15Rα-tdTr (5 × 10^6^ FFUs/100 μL PBS^−^), with 5 × 10^5^ MSCs/100 μL PBS^−^, or with 100 μL PBS^−^ (controls). In the two-dose therapy, mice were monitored for survival and sacrificed after 21 days (therapy endpoint). The lungs were excised, fixed in Bouin’s solution, and dissected into individual lobes. Visible melanoma foci were counted using a stereomicroscope. Peripheral blood and lung tissue were also used for flow cytometry studies. In the three-dose experiment, mice were monitored for survival and sacrificed after 21 days. Tumor tissues collected on days 2 and 9 after the last treatment (i.e., on days 14 and 21 of the experiment, respectively). The collected lung tissue was used to analyze by RT-qPCR the expression profile of several genes (encoding pro-inflammatory cytokines, CD4, and CD8, as well as PD-1 and PD-L1). Lung and liver tissues were examined by an experienced pathologist.

### Flow-Cytometric Analysis of NK and Tumor-Infiltrating Lymphocytes

Melanoma pulmonary foci were induced, and two-dose treatment was administered as described earlier. Mice (n = 3) were sacrificed, peripheral blood samples were collected in EDTA-coated tubes, and whole lungs were excised at 24-h and 48-h time points following initial treatment as well as after 21 days (for NK or CD4+ and CD8+ flow-cytometric analyses). Blood samples were treated with Red Blood Cell Lysis Buffer (BD Biosciences). Single-cell suspensions derived from lung tissue were obtained using digestion mix containing 0.5 mg/mL collagenase NB 4 (Nordmark Biochemicals), 0.2 mg/mL hyaluronidase type IV-S (Sigma-Aldrich), and 0.02 mg/mL DNase I (Worthington) in RPMI 1640 medium + 10% fetal bovine serum (FBS) and mashing the digest through a sterile 70-μm nylon mesh cell strainer into ice-cold PBS^−^ with 1% FBS. Red blood cells in lung digest were lysed (3 min) on ice using ACK Lysis Buffer (Lonza) and passed through a 40-μm nylon mesh cell strainer. To quantitate levels of NK, CD4+, and CD8+ cells, the generated samples were treated with antibodies (20 min at RT). After fluorescent labeling, 4 × 10^4^ cells were washed and analyzed using flow cytometry (BD FACSCanto). The following monoclonal antibodies were used (as per manufacturer’s instructions): PerCP/Cyanine5.5 anti-mouse CD45 (clone 30-F11; BioLegend), phycoerythrin (PE) anti-mouse CD3 (clone 17A2; BioLegend), allophycocyanin (APC) anti-mouse CD49b (to identify NK cells; clone DX5; eBioscences), fluorescein isothiocyanate (FITC) anti-mouse CD4 (clone GK1.5; BioLegend), and APC/Cyanine7 anti-mouse CD8a (clone 53-6.7; BioLegend).

### RNA Isolation and cDNA Synthesis

Total RNA was isolated from cultured infected and non-infected MSCs, as well as from lungs and liver frozen sections (n = 3) using the RNeasy Mini Kit (QIAGEN) according to the manufacturer’s instructions. The purity and concentration of RNA were measured spectrophotometrically with a Tecan Spark reader using a NanoQuant plate (Tecan). RNA was stored at −80°C. Synthesis of cDNA was performed from 100 ng total RNA using the High-Capacity cDNA Reverse Transcription Kit (Applied Biosystems) according to the manufacturer’s instructions. cDNA samples were further diluted 40-fold in nuclease-free water before further analysis.

### RT-qPCR Analysis of Gene Expression

RT-qPCR reactions were performed in duplicate for each sample using a reaction mix prepared as follows: 1× SYBR Select Master Mix (Applied Biosystems), 2 μL forward and reverse primers (1 μM each) (Table S1), and 4.0 μL of 40× diluted cDNA in a final volume of 15 μL. The amplification protocol included an initial preheating at 50°C for 2 min, initial denaturation at 95°C for 2 min, and 40 cycles of amplification (95°C for 15 s and 60°C for 60 s). Melting curve analyses were performed at the end of each run. RT-qPCR was carried out with a Rotor-Gene Q (QIAGEN, Hilden, Germany). Changes in the gene expression were rendered as a ratio of target gene versus reference gene (glyceraldehyde 3-phosphate dehydrogenase; GAPDH) relative to expression in control samples using the Pfaffl method,^57^ according to the following equation:

$$Ratio=\frac{{\left({\text{E}}_{target}\right)}^{\Delta Ct}{\text{Target}}^{\left(control-sample\right)}}{{\left({\text{E}}_{reference}\right)}^{\Delta Ct}{\text{Reference}}^{\left(control-sample\right)}},$$

where E represents the amplification efficiency, and Ct represents the number of PCR cycles needed for the signal to exceed a predetermined threshold value. Constitutive expression of B2M in MSC-vMyx-infected cells was rendered as a ratio of target gene (*B2M*) versus reference gene (*GAPDH*) relative to expression in control mock-infected MSCs using the Pfaffl method.^57^

### Histological and Immunohistochemical Assessments

Five-micrometer-thick, formalin-fixed, paraffin-embedded lung and liver sections were stained with standard H&E stain. Sections were scanned (3DHISTECH digital slide scanner) and analyzed using CaseViewer software (Fisher Scientific) by an experienced pathologist (E.C.).

### Immunofluorescence Staining of Tissues

Frozen OCT (optimal cutting temperature) blocks of tissues were cut using a cryotome into 5-μm-thick sections and fixed with paraformaldehyde (PAF) 4% at 15 min, 4°C. After incubation with 2% BSA for 30 min at RT, the slides were incubated overnight at 4°C with anti-F4/80 antibody (BM8, eBioscience) diluted 1:200, followed by incubation with anti-rat-488 (Abcam) for 1 h, and were then counterstained with DAPI. Negative controls were incubated with 2% BSA instead of primary antibodies. The stained tissue sections were imaged using a fluorescent confocal microscope.

#### Statistical Analysis

Data were expressed as means ± standard deviation (SD) as needed. Graphs were plotted using GraphPad Prism 7 (GraphPad Software). Statistical differences were determined using a one-way ANOVA test. Bartlett’s test was performed to ensure the suitability of the data for parametric significance tests. Kaplan-Meier survival curves were compared statistically using a log-rank test (Mantel-Cox). Significant differences in gene expression between groups in each sampling time were assessed by one-way ANOVA, followed by Tukey multiple comparison test in cases when the data were normally distributed or with the non-parametric Kruskal-Wallis test followed by Dunn’s test when the data were not. In the case of B2M, the t test was used to compare differences in constitutive expression of *B2M* between MSC mock-infected and MSC-vMyx-infected groups. Data are presented as bars indicating means ± SD. The significance levels are indicated with asterisks: ∗p ≤ 0.05; ∗∗p ≤ 0.01; ∗∗∗p ≤ 0.001. p values <0.05 were considered statistically significant.

### Ethics Approval

All procedures involving human bone marrow were approved by the Bioethics Committee of the Maria Skłodowska-Curie Memorial National Research Institute of Oncology, Warsaw, Poland (approval: 63/2017).

## Author Contributions

J.J.-R., A.S., and A.R. designed and performed *in vitro* and *in vivo* studies. G.M. and M.M.R. designed and provided MYXV recombinants. W.F. and A.H. isolated and characterized MSCs. W.F. and N.V. performed flow cytometry analyses. J.J.-R. and A.H. prepared viruses, performed cell culture, and performed RT-qPCR. E.C. analyzed histologic preparations. J.J.-R., A.S., A.R., G.M., and M.M.R. wrote/contributed to writing the manuscript. All authors read and approved the final manuscript.

## Conflicts of Interest

G.M. is a co-founder of OncoMyx Therapeutics, devoted to the clinical development of MYXV vectors for cancer. All authors declare no competing interests.

## Supplemental Information

Document S1. Table S1

Document S2. Article plus Supplemental information
