# Supplementary material for: Myxoma Virus-Loaded Mesenchymal Stem Cells in Experimental Oncolytic Therapy of Murine Pulmonary Melanoma
Source: Mol Ther Oncolytics. 2020 Jul 6;18:335–50. doi: 10.1016/j.omto.2020.07.003 (PMC7398944; doi:10.1016/j.omto.2020.07.003)
Supplement: Document S1. Table S1 [file mmc1.pdf]

OMTO, Volume *18*

Supplemental Information

Myxoma Virus-Loaded Mesenchymal Stem Cells in Experimental Oncolytic Therapy of Murine Pulmonary Melanoma

Joanna Jazowiecka-Rakus, Aleksander Sochanik, Aleksandra Rusin, Agata Hadryś, Wojciech Fidyk, Nancy Villa, Masmudur M. Rahman, Ewa Chmielik, Lina S. Franco, and Grant McFadden

Myxoma virus-loaded mesenchymal stem cells in experimental oncolytic therapy of murine pulmonary melanoma

Joanna Jazowiecka-Rakus, Aleksander Sochanik, Aleksandra Rusin, Agata Hadryś, Wojciech Fidyk, Nancy Villa, Masmudur M. Rahman, Ewa Chmielik, Lina S. Franco, Grant McFadden

Table S1. Primers

Gene name	Forward primer (5'-3')	Reverse primer (5'-3')
mPD-L1/ CD274	TGCGGACTACAAGCGAATCACG	CTCAGCTTCTGGATAACCCTCG
mINFɣ	CAGCAACAGCAAGGCGAAAAAGG	TTTCCGCTTCCTGAGGCTGGAT
mPD-1	CGGTTTCAAGGCATGGTCATTGG	TCAGAGTGTCGTCCTTGCTTCC
mIL-15	GTAGGTCTCCCTAAAACAGAGGC	TCCAGGAGAAAGCAGTTCATTGC
mCD8a	ACTACCAAGCCAGTGCTGCGAA	ATCACAGGCGAAGTCCAATCCG
mCD4	GTTCAGGACAGCGACTTCTGGA	GAAGGAGAACTCCGCTGACTCT
mTNF-α	GGTGCCTATGTCTCAGCCTCTT	GCCATAGAACTGATGAGAGGGAG
mIL-1β	TGGACCTTCCAGGATGAGGACA	GTTCATCTCGGAGCCTGTAGTG
mGAPDH	CATCACTGCCACCCAGAAGACTG	ATGCCAGTGAGCTTCCCGTTCAG
hB2M	CCACTGAAAAAGATGAGTATGCCT	CCAATCCAAATGCGGCATCTTCA
hGAPDH	GTCTCCTCTGACTTCAACAGCG	ACCACCCTGTTGCTGTAGCCAA

Multimedia File S1. **Co-culture of MYXV-infected MSCs with melanoma cells.** Time-lapse (3-48h) fluorescence microscopy of vMyx-EGFP-infected (MOI=10) MSCs (green fluorescence) co-cultured (1:2 cell/cell ratio) with mRFP-expressing B16-F10 (red fluorescence). Cell-to-cell contact (visible from ca. 3-h time point) between MSCs and melanoma cells enabling cross-infection; yellow-orange fluorescence (overlay) from infected melanoma cells visible besides red and green signals (magn. 10×, scale bar 50 μm); (AVI file: 97.4 MB).
